# Association between the time spent watching television and the sociodemographic characteristics with the presence of overweight and obesity in Colombian adolescents (secondary analysis of the ENSIN 2010)

**DOI:** 10.1371/journal.pone.0216455

**Published:** 2019-05-07

**Authors:** David Rincón-Pabón, Yeraldin Urazán-Hernández, Jhonatan González-Santamaría

**Affiliations:** ZIPATEFI—Zona de Investigaciones de Posgrados de la Andina, Fundación Universitaria del Área Andina. Pereira, Colombia; Cairnmillar Institute, AUSTRALIA

## Abstract

**Objective:**

To determine the association between the time spent watching television and the sociodemographic characteristics with the presence of overweight and obesity in Colombian adolescents.

**Materials and methods:**

Secondary analysis of the information obtained in the National Survey of the Nutritional Situation 2010 of Colombia, in a probabilistic sample of 18177 adolescents with an age range between 13 and 17 years.

The time spent watching television and / or videogames and sociodemographic factors was determined. Anthropometric markers and body composition were recorded. Associations were established through logistic regression models.

**Results:**

The prevalence of overweight was 13.0% (95% CI 12.4–13.8) and obesity was 3.0% (95% CI 2.8–3.3). The prevalences of overweight and obesity were higher in adolescent women [16.6% (95% CI 15.8–17.5) and 3.4% (95% CI 3.1–3.7), respectively].

Overweight and obesity were associated with being female [OR 1.94 (95%CI 1.77–2.12) and OR 1.29 (95%CI 1.09–1.53), respectively], high socioeconomic level [OR 1.22 (95%CI 1.10–1.36) and OR 1.47 (95%CI 1.19–1.81), respectively], reside in urban area [OR 1.13 (95%CI 1.03–1.24) and OR 1.47 (95%CI 1.21–1.79), respectively]. Being indigenous was associated with being overweight [OR 1.25 (95%CI 1.10–1.42)], while being Afro-Colombian was associated with obesity [OR 1.33 (95%CI 1.05–1.69)]. Watching television and / or video games for two or more hours a day [OR1.17 (95%CI 1.06–1.30)] was associated with being overweight; whereas watching television every day of the week was associated with obesity [OR 1.66 (95%CI 1.13–2.43)].

**Conclusions:**

The population studied has a significant prevalence of overweight and obesity, being overweight is associated with being a woman, a high socioeconomic status, residing in an urban area, having an indigenous ethnicity, watching television for three days during the week and looking at more than two hours of television in a day; Obesity is associated with being a woman, a medium-high and high socioeconomic status, residing in an urban area, Afro-Colombian ethnicity, watching television at least once in the last week and watching television every day during the last week.

## Introduction

The World Health Organization (WHO) defines overweight and obesity as an abnormal and excessive accumulation of fat that can be harmful to health, and is the result of energy imbalances between calories consumed and spent. It is considered that overweight and obesity are a chronic disease that represent the epidemic of the XXI century and are identified by the Body Mass Index (BMI), a simple indicator that relates weight and height (weight in kilograms divided by square of height in meters). According to the World Health Organization (WHO), the BMI provides the most useful measure of overweight and obesity, being determined with a BMI greater than or equal to 25, or greater than or equal to 30, respectively [1]. Overweight and obesity is favored by the existence of an obesogenic environment that is characterized by the extreme availability of rich, high-calorie foods with little nutritional value, the development of a sedentary type of leisure that includes consumption of countless hours of television a day, videogame consoles, smartphones and a significant decrease in the hours dedicated to the practice of physical activity, both in the form of sports and games [2].

A high BMI is an important risk factor for non-communicable diseases, such as cardiovascular diseases (mainly heart disease and stroke), which were the main cause of death in 2012; diabetes; disorders of the locomotor system (especially osteoarthritis, a degenerative joint disease) and some cancers (endometrium, breast, ovaries, prostate, liver, gallbladder, kidney and colon). The risk of contracting these non-communicable diseases increases with the increase in BMI [3].

According to WHO, overweight and obesity has increased progressively, from 4% in 1975 to over 18% in 2016 [3]. On the other hand, a report from WHO and PAHO (Pan American Health Organization) [4], in Latin America, between 20% and 25% of children under 19 are affected by overweight and obesity. The rates vary in the adolescent population from 17% (Colombia) to 35% (Mexico).

Currently, and according to data from the National Survey of the Nutritional Situation 2010 of Colombia (ENSIN), the prevalence of overweight and obesity in children and adolescents has increased by 25.9% in the last five years and 62% of this population, watch television and / or play video games for two hours or more [5]. In addition, according to the ENSIN, one of every 6 children and adolescents is overweight or obese; this relationship increases as the SISBEN level increases. Based on this, the present study aims to determine the association between the time spent watching television and the sociodemographic characteristics with the presence of overweight and obesity in adolescents in Colombia, in order to corroborate the research hypothesis which is that watching television and / or playing video games for prolonged periods is associated with the presence of overweight and obesity in Colombian adolescents.

## Materials and methods

### Population

It is a secondary descriptive and transversal analysis of the information obtained in the National Nutrition Situation Survey 2010, which was funded by the Colombian Family Welfare Institute (ICBF) and carried out during the years 2008 to 2010 in 18177 adolescents of ages between 13 and 17 years old. This survey is a cross-sectional measurement, carried out to determine the prevalence of nutritional problems and some health conditions in the Colombian population [5].

### Sampling

The sample for the ENSIN (2010) was probabilistic, of conglomerates (groupings "natural" relatively homogeneous in a population), stratified (the entire population was divided into different subgroups or subpopulations) and multistage (consisted in taking samples by stages using units of descending sampling in order to make the process more practical). The study group was constituted by 99% of the population residing in private homes in the urban and rural areas. A total of 50,670 households were studied, including a population of 258 municipalities or primary sampling units (UPM) of the 32 departments of the Country and Bogotá D.C. The segments were proportional in the municipal capitals and in the rural area, and were concentrated in 1920 aggregates or subgroups [5].

The compilation of the information was face to face and by a professional in Nutrition and Dietetics, trained in the data collection instruments, and use of PDA personal digital assistants. During the training, the following aspects were highlighted: The procedures to conduct an interview, establish a good relationship with the interviewee, and make a good first impression. The ethical conditions of the interview such as neutrality, confidentiality, not suggesting answers, not making value judgments, not changing the construction or sequence of questions, not creating stereotypes, and not rushing the interview [5].

### Data collection

The instrument for data collection was developed by the ICBF and was the same questionnaire applied in the 2005 ENSIN. The questions were addressed to the adolescent or to a person of the household who could provide reliable information [5].

The time spent watching television and / or playing video games was defined taking into account the following question addressed to the adolescent: During the last 7 days, did you watch television or play video games? Those who responded in a positive way, were asked about the frequency of the event through the question: How many days did you watch television or play video games in the last seven days? followed by: In those days, how much time did you spend watching television or playing video games? In the case that the time reported presented variations between days, the question was asked: In the last 7 days, how much time in total did you watch television or play with video games? [5].

Emphasis was placed on the sum of the time of watching television and using video games, which could be at home or in an external place destined for that purpose. The pollsters always carried a calculator. Only the temporary reference of the last 7 days (calendar days) was taken into account. In case the adolescent did not report the average per day, but day by day, an arithmetic average was made with a calculator to establish the average per day. If it is not possible to calculate the average day because the individual did not remember one day, the question that was asked was about the total time in which the activity was carried out. The purpose of establishing an average of watching television in the last seven days is to determine the average time that adolescents watch television, since there is a significant variation in the different days (weekdays and weekends).[5]

The anthropometric data were obtained directly; the weight was measured with a digital scale (SECA Model 872) with a capacity of 200 kilos and an accuracy of 50 grams. Each balance was adjusted to compensate for the gravitational attraction that corresponds to the geographic latitude of Colombia (between 0 and 15 degrees north latitude), the subjects wore very light clothing and no shoes; the height was measured with a portable wooden stadiometer, which consisted of a main piece and two extensions with a capacity to measure up to 2 meters, with sensitivity of 1 millimeter. Also, it has a movable piece that serves as a stop for the head and is measured standing up. People measured themselves without shoes, braids, hairstyles, ornaments or other objects on the head that could interfere with the measurement. The stadiometer complied with the technical specifications, modified by UNICEF-CENAN; and they had a quality control certificate and code number, signed by a UNICEF anthropometric consultant [5].

All the questions that were applied in the questionnaire were read aloud and literally to each person surveyed without any interpretations or clarifications of any kind by the interviewer. When the respondent did not understand any of the questions, even though the interviewer repeated it verbatim, no clarifications were given and the questions of the following domain were passed on [5].

The study was approved by the ethics committee of the National Nutrition Situation Survey [5].

### Definitions

Overweight and obesity (nominal binary variables) were defined through the nutritional classification given by Z score for the Body Mass Index (BMI) for age. The cut-off point was defined as values> 1 to ≤ 2 standard deviations for overweight and values> 2 standard deviations for obesity [6].

Likewise, the following sociodemographic variables were defined: Age (binary variable): between 13 and 14 years old and between 15 and 17 years old; sex (binary variable): male and female; the socioeconomic level (ordinal variable) was determined according to the identification system of possible beneficiaries of social programs in Colombia-SISBEN III (1 to 4), with level 1 being the one with the highest poverty and level 4, the least or no poverty, said classification is a measure developed by the Department of National Planning of Colombia, which takes into account sociodemographic characteristics, living conditions and access to public services; area or origin (binary variable): urban or rural; Ethnicity (nominal variable) was established based on belonging to an ethnic group by self-recognition (Afro-Colombian for those subjects descending from the African diaspora and indigenous for subjects of Native American descent); and exposure to television and / or video games: Depending on the answers given by the informants, the adolescents were classified into two categories: less than 2 hours a day and 2 hours a day or more. This cut-off point has been defined by the American Academy of Pediatrics as the criterion for classifying those subjects who have or do not use excessive television or video games [7], [8]. “Table 1”

**Table 1:** List of variables that were measured in the survey.

**Age**
13 to 14 years
15 to 17 years
**Sex**
Male
Female
**Socioeconomic level**
Level i
Level ii
Level iii
Level iv or more
**Geographic area**
Urban
Rural
**Ethnicity**
NONE
Afrocolombian
Indigenous
**Watched television and / or videogames in the last 7 days**
Yes
No
**Number of days that you watched television and / or video games in the last week**
None
One day
Two days
Three days
Four days
Five days
Six days
Seven days
**Number of hours of watching TV or video games in the day**
Less then two days
2 hours or more

### Statistic analysis

The nominal variables were expressed as frequencies. Comparison of proportions was performed with Chi-square tests (X2) to test the statistical significance. Subsequently, an exploratory analysis was conducted to determine the percentage distribution for each of the associated factors. To estimate the relationship between overweight-obesity, sociodemographic variables and time spent watching television and / or video games was assessed using crude prevalence odds ratios (OR) and a multivariable (adjusted) logistic regression model. The binary logistic regression model was adjusted for the age variable and the second model was adjusted for all the variables. The analyzes were performed in the Statistical Package for Social Science software, version 20 (SPSS, Chicago, IL, United States), and a p value <0.05 was considered significant.

## Results

The study was constituted by 7718 adolescents from 13 to 14 years old (42.5%) and 10459 from 15 to 17 years old (57.5%), for a total of 18177 adolescents, 9044 were men (49.8%) and 9133 women (50.2%). Of the total population studied, 16777 adolescents watched television and / or video games in the last week (92.3%), 4892 adolescents watched TV less than two hours in a day (29.2%) and 11870 adolescents watched TV two or more hours in a day (70.8%). No Significant differences were found in the percentage composition of age and socioeconomic level “Table 2”.

**Table 2:** Characteristics of study participants among adolescents aged 13–17 years in Colombia.

	Total n (%)	Male n (%)	Female n (%)	p-Value
All of the participants	18177 (100)	9044 (49.8)	9133 (50.2)	
**Age**				
13 to 14 years	7718 (42.5)	3860 (42.7)	3858 (42.2)	0.550
15 to 17 years	10459 (57.5)	5184 (57.3)	5275 (57.8)	
**Socioeconomic level**				
Level i	10376 (57.1)	5180 (57.3)	5196 (56.9)	0.693
Level ii	2207 (12.1)	1108 (12.3)	1099 (12.0)	
Level iii	1629 (9.0)	816 (9.0)	813 (8.9)	
Level iv or more	3965 (21.8)	1940 (21.5)	2025 (22.2)	
**Geographic area**				
Urban	12265 (67.5)	5963 (65.9)	6302 (69.0)	0.000
Rural	5912 (32.5)	3081 (34.1)	2831 (31.0)	
**Ethnicity**				
None	14050 (77.3)	6947 (76.8)	7096 (77.7)	0.048
Afrocolombian	2082 (11.5)	1081 (12.0)	1001 (11.0)	
Indigenous	2045 (11.3)	1015 (11.2)	1030 (11.3)	
**Watched television and / or videogames in the last 7 days**				
Yes	16777 (92.3)	8387 (92.7)	8375 (91.7)	0.007
No	1400 (7.7)	648 (7.2)	752 (8.2)	
**Number of days that you watched television and / or video games in the last week**				
None	1415 (7.8)	657 (7.3)	758 (8.3)	0.000
One day	500 (2.8)	271 (3.0)	229 (2.5)	
Two days	795 (4.4)	416 (4.6)	379 (4.1)	
Three days	927 (5.1)	524 (5.8)	403 (4.4)	
Four days	712 (3.9)	358 (4.0)	354 (3.9)	
Five days	1196 (6.6)	523 (5.8)	673 (7.4)	
Six days	984 (5.4)	438 (4.8)	546 (6.0)	
Seven days	11648 (64.1)	5857 (64.8)	5791 (63.4)	
**Number of hours of watching TV or video games in the day**				
Less then two days	4892 (29.2)	2555 (30.5)	2337 (27.9)	0.000
2 hours or more	11870 (70.8)	5832 (69.5)	6038 (72.1)	

Statistical significance <0.05 with Chi-square tests.

The overall prevalence of overweight was 13.0% (95%CI 12.4–13.8). There was a significant difference in prevalences between men and women, with a higher prevalence of obesity among adolescent women [16.6% (95%CI 15.8–17.5)]. Regarding socioeconomic level, significant differences were observed in the prevalence of overweight, with a high prevalence in the group with the highest socioeconomic level or level IV or higher [15.0% (95%CI 13.7–16.3)]. The geographic area showed differences in the prevalence of overweight, with the prevalence of overweight in the urban group being higher [13.4% (95%CI 12.8–14.0)]. Likewise, differences of prevalence among ethnic groups were observed, with the indigenous ethnic group having the highest prevalence of overweight [15.5% (95%CI 14.0–18.1)]. (Table 3). The prevalences of overweight also showed differences in the number of days that the adolescent watched television and / or video games in the last week, being higher in the young people who watched television for four and seven days [13.8% (95%CI 10.4–14.0) and 13.4% (95%CI 12.7–14.2), respectively], also showed differences in prevalence in the number of hours that a teenager watches television and / or video games in a day, showing a higher prevalence of overweight those who watch television two or more hours per day [13.5% (95%CI 13.0–14.3)]. “Table 4”.

**Table 3:** Prevalence of overweight and obesity, and sociodemographic factors in a representative sample of colombian adolescents.

	Overweight			Obesity		
	n	% (95% CI)	p-Value	n	% (95% CI)	p-Value
All of the participants	2358	13.0 (12.4–13.8)		546	3.0 (2.8–3.3)	
**Age**						
13 to 14 years	1003	13.0 (12.0–14.0)	0.936	228	3.0 (2.6–3.6)	0.736
15 to 17 years	1355	13.0 (12.2–13.6)		318	3.0 (2.8–3.3)	
**Sex**						
Male	841	9.3 (8.6–9.8)	0.000	238	2.6 (2.3–3.0)	0.003
Female	1517	16.6 (15.8–17.5)		308	3.4 (3.1–3.7)	
**Socioeconomic level**						
Level i	1301	12.5 (11.5–12.9)	0.000	258	2.5 (2.1–2.8)	0.000
Level ii	261	11.8 (10.2–13.1)		73	3.3 (2.7–4.5)	
Level iii	203	12.5 (11.2–14.8)		72	4.4 (3.4–5.6)	
Level iv or more	593	15.0 (13.7–16.3)		143	3.6 (3.0–4.5)	
**Geographic area**						
Urban	1646	13.4 (12.8–14.0)	0.010	410	3.3 (3.0–3.7)	0.000
Rural	712	12.0 (11.1–12.9)		136	2.3 (2.0–2.7)	
**Ethnicity**						
None	1791	12.8 (12.3–13.3)	0.003	430	3.1 (2.7–3.3)	0.000
Afrocolombian	249	12.0 (10.4–13.0)		84	4.0 (3.2–4.6)	
Indigenous	317	15.5 (14.0–18.1)		32	1.6 (0.9–2.2)	

Statistical significance <0.05 with Chi-square tests.

**Table 4:** Prevalence of overweight and obesity, and exposure to television and / or video games in a representative sample of colombian adolescents.

	Overweight			Obesity		
	n	% (IC 95%)	p-Value	n	% (IC 95%)	p-Value
All the participants	2358	13.0 (12.4–13.8)		546	3.0 (2.8–3.3)	
**Watched television and / or videogames in the last 7 days**						
Yes	2182	13.0 (12.5–13.6)	0.434	517	3.1 (2.8–3.3)	0.033
No	172	12.3 (11.2–14.3)		29	2.1 (0.9–3.0)	
**Number of days that you watched television and / or video games in the last week**						
None	176	12.4 (10.2–15.2)	0.035	29	2.0 (1.3–2.6)	0.031
One day	61	12.2 (9.5–15.5)		12	2.4 (1.2–4.2)	
Two days	99	12.5 (10.2–16.7)		18	2.3 (1.0–3.7)	
Three days	85	9.2 (7.3–11.3)		22	2.4 (1.6–3.5)	
Four days	98	13.8 (10.4–16.4)		14	2.0 (.6–3.2)	
Five days	150	12.5 (10.1–14.0)		36	3.0 (1.9–3.6)	
Six days	131	13.3 (10.4–15.2)		26	2.6 (1.6–4.0)	
Seven days	1558	13.4 (12.7–14.2)		389	3.3 (3.0–3.7)	
**Number of hours of watching TV or video games in the day**						
Less then two days	576	11.8 (9.9–13.2)	0.002	139	2.8 (2.3–3.3)	0.243
2 hours or more	1606	13.5 (13.0–14.3)		378	3.2 (2.9–3.5)	

Statistical significance <0.05 with Chi-square tests.

The overall prevalence of obesity was 3.0% (95%CI 2.8–3.3). There was a significant difference in prevalences between men and women, with a higher prevalence of obesity among adolescent women [3.4% (95%CI 3.1–3.7)]. Regarding the socioeconomic level, significant differences were observed in the prevalence of obesity, evidencing a higher prevalence of obesity in the groups of levels III and IV or more [4.4% (95%CI 3.4–5.6) and 3.6% (95%CI 3.0–4.5), respectively]. The geographic area showed differences in the prevalence of obesity, with a higher prevalence of obesity in the urban group [3.3% (95%CI 3.0–3.7)]. Similarly, differences in prevalence among ethnic groups were observed, with adolescents belonging to the Afro-Colombian ethnic group having the highest prevalence of obesity [4.0% (95%CI 3.2–4.6)]. (Table 3). The prevalences of obesity also showed differences in terms of seeing or not watching television in the last seven days, with the prevalence of obesity being higher in adolescents who watch television [3.1% (95%CI 2.8–3.3)]. Regarding the number of days that the adolescent watched television and / or video games in the last week, significant differences were observed, the prevalence of obesity was in the young people who watched television for five and seven days [3.0% (95%CI 1.9–3.6) and 3.3% (95%CI 3.0–3.7)]. “Table 4”.

In the unadjusted binary logistic regression, women were found to have 94% [OR 1.94 (95%CI 1.77–2.12)] more likely to be overweight than men, belonging to the high socioeconomic level was 22% [OR 1.22 (95%CI 1.10–1.36)] more likely to be overweight compared to those of low socioeconomic status, as well as residing in urban areas [OR 1.13 (95%CI 1.03–1.24)] and being indigenous [OR 1.25 (95%CI 1.10–1.42)] was associated with overweight. In terms of watching television and / or video games, the only variable that showed a significant association was watching television for two hours or more a day [OR 1.17 (95%CI 1.06–1.30)], showing that the subjects who in one day see two hours or more of television showed a probability of 1.17 times of being overweight. In the adjusted models (1 and 2) the same variables that the crude OR to be overweight remained significantly associated. “Table 5”.

**Table 5:** Socio-demographic factors and television exposure associated to overweight in a representative sample of adolescents in Colombia.

Risk factor		**Crude odds ratio**^a^		**Adjusted model 1**^b^		**Adjusted model 2**^c^	
		**OR (95% CI)**	**p-Value**^d^	**OR (95% CI)**	**p-Value**^d^	**OR (95% CI)**	**p-Value**^**d**^
**Age**	13 to 14 years	1		1		1	
	15 to 17 years	0.99 (0.91–1.08)	0.93	0.99 (0.91–1.08)	0.93	0.98 (0.89- 1.07)	0.65
**Sex**	Male	1		1		1	
	Female	1.94 (1.77–2.12)	0.00	1.94 (1.78- 2.13)	0.00	1.89 (1.72 -2.08)	0.00
**Socioeconomic level**	Level i	1		1		1	
	Level ii	0.93 (0.81–1.07)	0.35	0.94 (0.81–1.08)	0.36	0.98 (0.84 -1.13)	0.75
	Level iii	0.99 (0.84–1.16)	0.93	0.99 (0.85–1.16)	0.93	1.03 (0.87 -1.21)	0.74
	Level iv or more	1.22 (1.10–1.36)	0.00	1.23 (1.10- 1.36)	0.00	1.26 (1.13 -1.41)	0.00
**Geographic area**	Urban	1.13 (1.03–1.24)	0.01	1.13(1.03- 1.24)	0.01	1.14 (1.02 -1.26)	0.02
	Rural	1		1		1	
**Ethnicity**	None	1		1		1	
	Afrocolombian	0.92 (0.80–1.07)	0.30	0.93 (0.81- 1.07)	0.31	0.99 (0.85 -1.15)	0.89
	Indigenous	1.25 (1.10–1.42)	0.00	1.25 (1.10- 1.43)	0.00	1.43 (1.24 -1.67)	0.00
**Watched television and / or videogames in the last 7 days**	Yes	1.06 (0.90–1.26)	0.43	1.07 (0.91- 1.26)	0.43	1.12 (0.94 -1.33)	0.21
	No	1		1		1	
**Number of days that you watched television and / or video games in the last week**	None	1		1		1	
	One day	0.97 (0.71–1.33)	0.88	0.98 (0.72-1.33)	0.89	0.98 (0.70-1.39)	0.92
	Two days	1.00 (0.77–1.30)	0.99	1.00 (0.77-1.30)	0.99	0.70 (0.50-1.00)	0.05
	Three days	0.71 (0.54–0.93)	0.01	0.71 (0.54-0.93)	0.01	1.07 (0.76-1.51)	0.70
	Four days	1.12 (0.86–1.46)	0.38	1.12 (0.86–1.46)	0.39	0.93 (0.68-1.28)	0.66
	Five days	1.01 (0.80–1.27)	0.93	1.01 (0.80–1.27)	0.94	0.99 (0.71-1.37)	0.94
	Six days	1.08 (0.84–1.37)	0.52	1.08 (0.85–1.38)	0.53	1.01 (0.76-1.33)	0.95
	Seven days	1.08 (0.92–1.28)	0.32	1.09 (0.92-1.28)	0.33	^e^	
**Number of hours of watching TV or video games in the day**	Less then two days	1		1		1	
	2 hours or more	1.17 (1.06-1.30)	0.00	1.17 (1.06–1.30)	0.00	1.11 (1.00 -1.23)	0.05

a Crude Odds Ratio (Without adjusting for other variables).

b Model adjusted for age.

c Model adjusted for age, sex, socioeconomic level, geographic area, ethnic group and exposure to television and / or video games.

d Statistical significance <0.05

e Due to redundancies, the degrees of freedom have been reduced for one or more variables.

Regarding obesity, crude OR showed that this was significantly associated with being a woman [OR 1.29 (95%CI 1.09–1.53)], belonging to socioeconomic level II, III and IV or more [OR 1.34 (95%CI 1.03–1.75), OR 1.81 (95%CI 1.39–2.37) and OR 1.47 (95%CI 1.19–1.81), respectively], reside in urban area [OR 1.47 (95%CI 1.21–1.79)], be Afro-Colombian [OR 1.33 (95%CI 1.05–1.69)], watch television and / or video games in the last 7 days [OR 1.51 (95%CI 1.03–1.20)], watch TV and / or video games in the last week for seven days [OR 1.66 (95%CI 1.13–1.43)]. Watching television and / or video games in a day for two hours or more did not show association. In model 1, adjusted for age, the same variables of crude OR remained significantly associated with obesity. In the second model (adjusted for all variables), it was only evident that they were significantly associated with obesity: being a woman, belonging to socioeconomic level III and IV or more, residing in an urban area and belonging to the Afro-Colombian ethnic group. “Table 6”.

**Table 6:** Socio-demographic factors and television exposure associated to obesity in a representative sample of adolescents in Colombia.

Risk factor		**Crude odds ratio**^a^		**Adjusted model 1**^b^		**Adjusted model 2**^c^	
		**OR (95% CI)**	**p-value**^d^	**OR (95% CI)**	**p-valor**^d^	**OR (95% CI)**	**p-value**^d^
**Age**	13 to 14 years	1		1		1	
	15 to 17 years	1.03 (0.87–1.22)	0.74	1.03 (0.87–1.22)	0.74	1.01 (0.85–1.21)	0.89
**Sex**	Male	1		1		1	
	Female	1.29 (1.09- 1.53)	0.00	1.29 (1.09- 1.53)	0.00	1.24 (1.04–1.48)	0.02
**Socioeconomic level**	Level i	1		1		1	
	Level ii	1.34 (1.03–1.75)	0.03	1.34 (1.03–1.75)	0.03	1.23 (0.93–1.61)	0.15
	Level iii	1.81 (1.39–2.37)	0.00	1.81 (1.39–2.37)	0.00	1.62 (1.23–2.13)	0.00
	Level iv or more	1.47 (1.19–1.81)	0.00	1.47 (1.19–1.81)	0.00	1.31 (1.05–1.63)	0.01
**Geographic area**	Urban	1.47 (1.21–1.79)	0.00	1.47 (1.21–1.79)	0.00	1.30 (1.05–1.62)	0.02
	Rural	1		1		1	
**Ethnicity**	None	1		1		1	
	Afrocolombian	1.33 (1.05–1.69)	0.02	1.33 (1.05–1.69)	0.02	1.41 (1.10–1.81)	0.01
	Indigenous	0.50 (0.35–0.72)	0.00	0.50 (0.35–0.72)	0.00	0.58 (0.38–0.87)	0.01
**Watched television and / or videogames in the last 7 days**	Yes	1.51 (1.03–2.20)	0.03	1.51 (1.03–2.20)	0.03	1.34 (0.91–1.98)	0.14
	No	1		1		1	
**Number of days that you watched television and / or video games in the last week**	None	1		1		1	
	One day	1.18 (0.59–2.32)	0.64	1.18 (0.59–2.32)	0.64	0.86 (0.41–1.81)	0.70
	Two days	1.11 (0.61–2.01)	0.74	1.11 (0.61–2.01)	0.74	0.93 (0.45–1.89)	0.84
	Three days	1.16 (0.66–2.04)	0.60	1.16 (0.66–2.04)	0.60	0.73 (0.33–1.60)	0.43
	Four days	0.96 (0.50–1.83)	0.90	0.96 (0.50–1.83)	0.90	1.10 (0.56–2.14)	0.78
	Five days	1.48 (0.90–2.43)	0.12	1.48 (0.90–2.43)	0.12	0.94 (0.47–1.89)	0.86
	Six days	1.30 (0.76–2.22)	0.34	1.30 (0.76–2.22)	0.34	1.18 (0.66–2.13)	0.58
	Seven days	1.66 (1.13–2.43)	0.01	1.66 (1.13–2.43)	0.01	^e^	
**Number of hours of watching TV or video games in the day**	Less then two days	1		1		1	
	2 hours or more	1.12 (0.92–1.37)	0.24	1.12 (0.92–1.37)	0.24	1.03 (0.84–1.26)	0.76

a Crude Odds Ratio (Without adjusting for other variables).

b Model adjusted for age.

c Model adjusted for age, sex, socioeconomic level, geographic area, ethnic group and exposure to television and / or video games.

d Statistical significance <0.05

e Due to redundancies, the degrees of freedom have been reduced for one or more variables.

## Discussion

According to the World Health Organization (WHO) and the Pan American Health Organization (PAHO), between 20% and 25% of children under 19 years of age are affected by overweight and obesity[9]. According to the findings of this study, the combined prevalence of overweight and obesity in Colombian adolescents between the ages of 13 and 17 years was 16% (13% and 3%, respectively). Prevalence that is significantly lower than that reported in Peru [10] and in the national health and nutrition surveys of Ecuador[11], Argentina[12] and Mexico[13]. However, the results of overweight in Colombia show a increase of more than two percentage points compared to the 2005 data (14). On the other hand, the results of the surveys of the mentioned countries are similar to the results of this study, regarding the differences in the prevalence of overweight and obesity, which are higher in the female sex.

In the present study, the high socioeconomic level of adolescents shows a higher prevalence of overweight and obesity than those of a lower socioeconomic level. This finding is consistent with studies conducted in Chile[14] and Argentina[15]; but contrary to that reported by Spain[16] in which they reported a lower prevalence of overweight and obesity in the highest socioeconomic level. This may happen because of socioeconomic variations between high and middle income countries, diversity in socio-cultural behavior and, especially, the inappropriate and prolonged use of new technologies[17–19]. In spite of the differences in prevalence, it should be taken into account that a higher socioeconomic level is associated with greater probabilities of overweight and obesity[20]. It is considered that food imbalances cause serious biological damage and its impact, measured by the burden of morbidity, is greater in middle-income countries[21]. According to a study[14], the probability of being overweight increases when belonging to the indigenous community, this result is similar to that found in this study, which found that indigenous people are approximately 25% more likely to develop Obesity than people with no ethnicity.

According to the most recent studies that the American Association of Pediatrics (AAP) suggest, establishing limits to watch television between 1 and 1.5 hours per day, can be more effective in preventing obesity than the standard of 2 hours per day [14]. this is reflected in the results of this study, which showed that adolescents who watched television and / or played video games for less than two hours a day had a lower prevalence of overweight and obesity compared to subjects with the same exposures or older than two hours of television.

In the present study, adolescents who watch 2 or more hours of television per day are 1.1 to 1.17 times more likely to be overweight than those with less than 2 hours; adolescents who watch television or play video games in the last week, are 1.66 times more likely to be obese every day than those with less than seven days. Overweight and obesity showed common associations in terms of high socioeconomic status, being a woman, residing in urban areas in adjusted models. These results are similar to those reported by Colombian data in 2005 (14), and in other countries such as France[22], Bangladesh[23] and Pakistan[24]. Possibly, the above results are due to the fact that overweight and obesity are consistent with a reduction in physical activity and an increase in the consumption of products with high caloric content and low nutritional value while watching television[25]. On the other hand, the previous study confirms that the consumption of food increases during the time that television is watched and goes hand in hand with unhealthy foods advertised in it, 54% of advertising spots are unhealthy foods[26].

When comparing the prevalence results of this study with data from Latin American and European studies, Colombia had the lowest percentages of overweight and obesity in adolescents, despite this, nevertheless the official data of Colombia in 2005 and those of the present study, show that overweight and obesity in Colombian adolescents increased 5.7% between 2005 and 2010. One of the main strengths of this study is the use of data from the ENSIN. The ENSIN is a representative data source at a national level in Colombia; Therefore, reliable estimates of adolescents in Colombia were achieved [5]. The analytical sample used in the current study corresponds to the total of adolescents surveyed in the ENSIN 2010, which allows a detailed analysis. However, this study also had limitations, since there was no classification of the time spent watching television and / or playing video games on holidays and food patterns were not taken into account during screen time. It has been found that the time spent watching television and / or playing video games increases on holidays [27]. The ENSIN reported that young people aged 14 to 18 years (34.2%) consume fast meals weekly and this percentage increases as the level of SISBEN increases [5].

## Conclusion

In conclusion, there is a significant prevalence of overweight and obesity among adolescents in Colombia. For overweight, the associated sociodemographic factors were: being a woman, high socioeconomic status, residing in an urban area, being an Indian, watching television and / or video games for three days in the last week and watching two or more hours of television and / or video games in one day. On the other hand, obesity is associated with sociodemographic factors such as: being a woman, medium socioeconomic level, medium-high and high, being Afro-Colombian, watching television and / or playing video games in the last seven days and watching television and / or video games for seven days during the last week. By adjusting the models by age and by all variables, overweight and obesity showed the same significant associations although with different values, being a woman, socioeconomic level III, IV or more and residing in an urban area.

In searches of information made by the authors, it was observed that the present study shows the most current findings of overweight-obesity association with the time spent watching television and sociodemographic determinants in a representative sample of adolescents from Colombia. Overweight and obesity in adolescents could be addressed through the control of sociocultural behaviors, such as: the good use of new technologies; establish limits to watch television between 1 and 1.5 hours per day, especially on holidays; perform and / or increase the level of physical activity; decrease the consumption of products with high caloric content and low nutritional value, essentially, while watching television.
